# Dialysis for end stage renal disease financed through the Brazilian National Health System, 2000 to 2012

**DOI:** 10.1186/1471-2369-15-111

**Published:** 2014-07-09

**Authors:** Lenildo de Moura, Isaías Valente Prestes, Bruce Bartholow Duncan, Fernando Saldanha Thome, Maria Inês Schmidt

**Affiliations:** 1 Post-Graduate Studies Program in Epidemiology, School of Medicine, Federal University of Rio Grande do Sul, Porto Alegre, Brazil; 2 Technical Unit for Health risks, Noncommunicable Diseases and Mental Health, Pan-American Health Organization, Setor de Embaixadas Norte, Lote 19, CEP: 70.800-400 Brasília, DF, Brazil; 3 School of Medicine, Federal University of Rio Grande do Sul, Porto Alegre, Brazil

**Keywords:** End-stage renal disease, Prevalence, Incidence, Ethnicity, Dialysis

## Abstract

**Background:**

Chronic kidney disease has become a public health problem worldwide. Its terminal stage requires renal replacement therapy – dialysis or transplantation – for the maintenance of life, resulting in high economic and social costs. Though the number of patients with end-stage renal disease treated by dialysis in Brazil is among the highest in the world, current estimates of incidence and prevalence are imprecise. Our aim is to describe incidence and prevalence trends and the epidemiologic profile of end-stage renal disease patients receiving publically-financed dialysis in Brazil between 2000 and 2012.

**Methods:**

We internally linked records of the High Complexity Procedure Authorization/Renal Replacement Therapy (APAC/TRS) system so as to permit analyses of incidence and prevalence of dialysis over the period 2000-2012. We characterized temporal variations in the incidence and prevalence using Joinpoint regression.

**Results:**

Over the period, 280,667 patients received publically-financed dialysis, 57.2% of these being male. The underlying disease causes listed were hypertension (20.8%), diabetes (12.0%) and glomerulonephritis (7.7%); for 42.3%, no specific cause was recorded. Hemodialysis was the therapeutic modality in 90.1%. Over this period, prevalence increased 47%, rising 3.6% (95% CI 3.2% - 4.0%)/year. Incidence increased 20%, or 1.8% (1.1% – 2.5%)/year. Incidence increased in both sexes, in all regions of the country and particularly in older age groups.

**Conclusions:**

Incidence and prevalence of end-stage renal disease receiving publically-financed dialysis treatment has increased notably. The linkage approach developed will permit continuous future monitoring of these indicators.

## Background

Chronic kidney disease has become a global public health problem. The terminal stage of this condition, end stage renal disease (ESRD), is a serious health outcome which carries high economic and social costs, requiring renal replacement therapy in the form of dialysis or transplantation in order to sustain life. The number of people with end-stage renal disease starting renal replacement therapy has increased worldwide in recent years, with a progressive increase in the proportion being elderly [1,2]. In high-income countries, end-stage renal disease accounts for a substantial share of health care spending and is one of the major contributors to rising health care costs. The annual increase in spending for dialysis programs around the world ranged from 6% to 12% over the last two decades and continues to grow, particularly in low- and middle-income countries [3].

The increase in the number of individuals globally with chronic kidney disease has stimulated the implementation of public policies to address this condition. These in turn require surveillance strategies designed to capture information relevant to prevention and treatment. National, local, or regional registries which collect and disseminate information on end-stage renal disease are available in some countries. In Brazil, expenses related to publically-financed renal replacement therapy are reimbursed through the High Complexity Procedure Authorization/Renal Replacement Therapy (Autorização de Procedimentos de Alta Complexidade/Terapia Renal Substitutiva, or APAC/TRS) subsystem of the Outpatient Information System (Sistema de Informação Ambulatorial, or SIA) [4]. APAC/TRS receives and records data for the approximately 84.9% of dialysis patients who are receiving treatment financed through Brazil’s national health system (Sistema Único de Saúde, or SUS) [5]. However, it has not been possible, to date, to regularly monitor the incidence, prevalence, and mortality of end-stage renal disease. Recently, we have created such a unified database by linking patient records within this APAC subsystem [4]. The aim of this study is to describe, using this linked database, trends in incidence and prevalence and the epidemiological profile of patients with end-stage renal disease receiving publically financed dialysis in Brazil between the years 2000 to 2012.

## Methods

We evaluated all patients with end stage renal disease undergoing dialysis financed through the SUS between January 1, 2000, and December 31, 2012, applying deterministic and probabilistic record linkage techniques to existing data files received from the Ministry of Health, as has been previously described [4].

We defined a case of chronic end stage renal disease as an individual for whom reimbursement was solicited via APAC/TRS for a minimum of three months, thus minimizing the possible inclusion of patients with acute renal failure not requiring chronic dialysis [6].

We produced descriptive analyses of dialysis patients sex, age, race/color (as ethnicity is officially characterized in Brazil), region of residence, underlying disease, and treatment modality. The race/color “brown”, or “pardo” in Portuguese, permits individuals to self-identify as of a mixed race, “black” referring basically to those who self-identify as being predominantly of African ancestry; “yellow” refers to those of Asian background, these predominantly being of Japanese ancestry [7]. To define the underlying disease that led to end stage renal disease we used a classification consisting of six groups based on codes from the International Statistical Classification of Diseases and Related Health Problems - Tenth Revision (ICD-10): diabetes mellitus, hypertension, glomerulonephritis, chronic interstitial nephritis, other diseases, and end-stage renal disease of uncertain cause [6]. Those with missing ICD-10 codes were excluded from this specific analysis.

Incidence and prevalence were expressed per 1,000,000 inhabitants/year (patients per million population, or pmp), using population data from the 2000 and 2010 demographic censuses and official interpolations for intercensus years [8]. As we could not distinguish new from prevalent cases in 2000, we defined incident cases from 2001 onwards.

We used Joinpoint regression for analysis of trends in the incidence of end-stage renal disease [9]. This regression analysis identifies the point at which statistically significant changes in tendencies occur over time, and then expresses the annual percent change (APC) and its 95% confidence interval over each separate temporal segment identified. Changes in trends may be either in magnitude or direction. A negative sign indicates a decline over a temporal segment.

Basic analyses were performed using Statistical Analysis System - SAS. Microsoft Excel was used for the calculation of incidence and prevalence. This study was approved by the Ethics in Research Committee of the Hospital de Clínicas de Porto Alegre on 10/03/2010 (No. 100056). As analyses are based on surveillance databases from the Ministry of Health, no patient consent was necessary. All those with access to data signed agreements to maintain the confidentiality of the information.

## Results

Over the period studied, a total of 280,667 patients with end-stage renal disease received publically-financed dialysis for at least 3 consecutive months. Of these 160,569 (57.2%) were males and 120,098 (42.8%) females. As Table 1 shows, most (72.6%) cases were treated in the southeast and northeast regions. The predominant age grouping was 45-64 years (43.4% of patients), with 29.9% of those treated being <44 years old, and 26.8% ≥ 65. In terms of race/color categories, for which information was available from 2008 on, 45.2% were registered as white, 9.7% as black, 30.8% as brown, 0.8% yellow (Asian) and 0.2% native Brazilian. After excluding 10924 cases with missing ICD-10 codes, the principal underlying causes of renal failure were hypertension (20.4%), diabetes mellitus (12.0%) and glomerulonephritis (7.7%). For 42.3% of patients, the cause was listed as “undetermined”. The predominant therapeutic modality (90.1%) was hemodialysis.

**Table 1:** Demographic and clinical characteristics of the 280,667 patients with chronic end-stage renal disease receiving publically-financed dialysis covering a period of at least 3 consecutive months

**Characteristic**	**Total**		**Males**		**Females**	
	**n**	**%**	**n**	**%**	**n**	**%**
Geographic region*						
North	11874	4.2	6791	4.2	5079	4.2
Northeast	59688	21.3	34867	21.7	24799	20.7
Southeast	144242	51.4	82057	51.1	62134	51.8
South	46322	16.5	26145	16.3	20161	16.8
Center-west	18538	6.6	10709	6.7	7822	6.5
Total	280664	100	160569	100	119995	100
Age (years)*						
0 - 19	9607	3.4	5176	3.2	4430	3.7
20 - 44	74163	26.5	39975	24.9	34188	28.5
45 - 64	121584	43.4	71487	44.6	50097	41.8
65 - 74	48175	17.2	27910	17.4	20265	16.9
75 e +	26811	9.6	15902	9.9	10909	9.1
Total	280340	100.0	160450	100	119889	100
Race/skin color**						
White	46014	45.2	27026	45.8	18988	44.3
Black	9897	9.7	5556	9.4	4341	10.1
Brown	31386	30.8	18100	30.7	13286	31.0
Yellow (Asian)	835	0.8	493	0.8	342	0.8
Native Brazilian	199	0.2	110	0.2	89	0.2
Lacking information	13484	13.2	7669	13.0	5815	13.6
Total	101815	100	58954	100	42861	100
Underlying disease*						
Undetermined	118349	42.3	68014	42.5	50335	42.1
Diabetes	33692	12.0	18466	11.5	15226	12.7
Hypertension	57166	20.4	33440	20.9	23726	19.8
Glomerulonephritis	21459	7.7	12172	7.6	9287	7.8
Chronic interstitial nephritis	7308	2.6	4321	2.7	2987	2.5
Other	41747	14.9	23671	14.8	18076	15.1
Total	279721	100	160084	100	119637	100
Therapeutic modality*						
Hemodialysis	250183	90.1	144992	91.2	105095	88.5
Peritoneal dialysis	27648	9.9	13960	8.8	13681	11.5
Total	277831	100	158952	100	118776	100

Brazil, 2000 to 2012.

*Small diferences in totals are due to missing data for specific variables.

**Data available from 2008 onward.

Table 2 shows the annual increase in prevalence of patients in dialysis from 2000 to 2012. Joinpoint regression indicated an average annual increase of 3.6% (95% CI 3.2% – 4.0%). The average annual increase in incidence was more discrete – 1.8% (1.1% – 2.5%)/year.

**Table 2:** Prevalence and incidence of publically-financed dialysis for end stage renal disease covering at least three consecutive months

		**Prevalence**			**Incidence**		
**Year**	**Population**	**n**	**Coefficient (pmp)**	**Annual change (%)**	**n**	**Coefficient (pmp)**	**Annual change (%)**
2000	169799848	57105	336.3		_		
2001	171705916	62891	366.2	8 .9	15788	91.9	
2002	173704941	65324	376.0	2 .7	15859	91.2	-0 .8
2003	175730799	70619	401.8	6 .9	17210	97.9	7 .3
2004	177783157	74083	416.7	3 .7	17712	99.6	1 .7
2005	179863161	78371	435.7	4 .6	18091	100.5	0 .9
2006	181971226	82265	452.0	3 .7	18395	101.0	0 .5
2007	184107444	85851	466.3	3 .2	18691	101.5	0 .5
2008	186272178	85521	459.1	-1 .5	17530	94.1	-7 .3
2009	188466163	91361	484.7	5 .6	19706	104.5	11 .1
2010	190755799	95918	502.8	3 .7	20691	108.4	3 .7
2011	192943315	100614	521.4	3 .7	21885	113.4	4 .6
2012	193976530	104433	538.3	3 .2	22004	113.4	0 .0

Brazil, 2000 to 2012.

Sources: APAC; Instituto Brasileiro de Geografia e Estatística – IBGE (4).

pmp = patients per 1 million population.

Trends in the incidence of dialysis, expressed as the annual variation in incidence as estimated by the Joinpoint software, are presented by geographical region and sex in Table 3. One notes an increase in incidence in both men and women in all regions of the country, although slightly less so in the south, where the increase among males was 1.2% (0.2% – 2.2%) and among females 0.2% (-0.6% – 1.0%). Except in the north region, the increase was always greater in males. When the estimated trend varied in a statistically significant way over the period, as occurred in the north region, the trend in each temporal segment is shown.

**Table 3:** Trends in the incidence of publically-financed dialysis for end stage renal disease covering a minimum of 3 consecutive months, by geographic region and sex

**Region**	**Males**		**Females**	
	APC*	**(IC 95%)**	APC*	**(IC 95% )**
**Brazil**	2.1	(1.4-2.9)	1,3	(0.6- 2.1)
**North**	3.8	1.5 – 6.2)	3.8	(2.0 – 5.6)
**Northeast**	4.1	(3.6 – 4.7)	3.5	(2.6 – 4.4)
**Southeast**	1.4	(0.7 – 2.1)	0.6	(-0.2 – 1.4)
**South**	1.2	(0.2 – 2.2)	0.2	(-0.6 – 1.0)
**Center-west**	3.5	(2.4 – 4.6)	2.6	(1.4 – 3.8)

Brazil, 2001 to 2012.

*Annual percentage change (APC).

Similarly, specific trends for different age/sex groupings are shown in Table 4. Incidence fell over the period for younger Brazilians (ages 0-19 and 20-44). For 0-19 year old males, the decline was -2.3% (-3.5% – 1.1%)/year, and for men 20-44, -1.1% (-1.8% – -0.3%)/year. For females the decline was -1.1% (-2.4% – 0.2%)/year and -0.8 (-1.5% – 0.0%), for the 0-19 and 20-44 age groups, respectively. Older men (45-64, 65-74 and 75+) presented annual increases of 0.9% (0.2% – 1.7%), 2.3% (1.6% – 3.0%) and 3.0% (2.0% – 4.1%), respectively. Incidence in women declined in the 45-64 age range, increased slightly for those between 65-74 and increased 2.2% (1.1% – 3.4%) for those 75 or over.

**Table 4:** Trends in the incidence of publically-financed dialysis for end stage renal disease covering a minimum of 3 consecutive months, by age and sex

**Age (years)**	**Males**		**Females**	
	APC*	**(IC 95%)**	**APC**	**(IC 95%)**
**0 – 19**	-2.3	(3.5 – -1.1)	-1.1	(-2.4 – 0.2)
**20 - 44**	-1.1	(-1.8 – -0.3)	-0.8	(-1.5 – 0.0)
**45 – 64**	0.9	(0.2 – 1.7)	-0.4	(-1.1 – 0.3)
**65 - 74**	2.3	(1.6 – 3.0)	0.4	(-0.6 – 1.4)
**75 +**	3.0	(2.0 – 4.1)	2.2	(1.1 – 3.4)

Brazil, 2001 to 2012.

*Annual percentage change (APC).

## Discussion

Our results, based on national data of renal dialysis in the national health system, estimated to cover 85% of Brazilian patients in dialysis, illustrate the growing importance of ESRD in Brazil. From 2000 to 2012, the prevalence of ESRD receiving dialysis increased by 46.8%, an average of 3.6% per year, and the incidence by 20%, an average of 1.8% per year. By 2011, the most recent year with complete ascertainment, a total of 521.4 cases pmp existed, of which 113.4 cases pmp had initiated treatment that year.

To date, considerable uncertainty has existed as to incidence and prevalence of patients with ESRD undergoing dialysis in Brazil. Frequently cited data have come from an annual query of the Brazilian Nephrology Society to its members. However, as reporting through this query is voluntary and incomplete, with only 55% of dialysis centers responding in 2011, uncertainty regarding the exact numbers remains. In fact, our APAC prevalence estimate for 2011 (521 pmp), is greater than the Society’s (475 pmp) [5]. The true difference is even greater, as APAC covers only publically financed treatment, roughly 85% of all dialysis, and our analyses excluded patients on dialysis for less than 3 months [5]. Further, in terms of estimating total cases of ESRD, neither the frequencies here reported, nor those of the Brazilian Nephrology Society include patients who die of ESRD before receiving renal replacement therapy or patients undergoing transplantation without previous dialysis for at least three months.

In any event, by international comparison, a massive number of patients receive dialysis in Brazil. Grassmann et al, in their global overview of ESRD, placed Brazil among countries with the greatest number of patients receiving such treatment in 2004 [2]. The U.S. Renal Data System (USRDS), which estimated a prevalence of renal dialysis of 679 pmp in 2011 for Brazil, ranked the country third in the world for that year in terms of numbers of patients undergoing dialysis [10].

This position is in large part due to the size of the population, as prevalence rates are at the low end of the spectrum shown in the USRDS’s international comparisons. Approximately half of the countries listed by the USRDS, in general high income countries, had prevalence rates of >1000 pmp. The relatively lower prevalence in Brazil may well reflect remaining problems of access to therapy. According to a recent publication of the Latin American Dialysis and Renal Transplant Registry (RLDTR), prevalence and incidence are increasing across the region. Several South American countries have dialysis prevalence in 2008 - Argentina (620 pmp) Chile (852 pmp) and Uruguay (825 pmp) - greater than those we report (459 pmp) for that year, with the overall prevalence for the region (461 pmp) being quite similar to ours [11]. Of note, however, these comparisons are not adjusted for age, and many of the countries with higher rates of dialysis, especially the high income countries, have a more elderly population. The prevalence of ESRD treated by dialysis in Brazil is likely to continue to increase, since the transplantation rate is around 26 pmp/year in Brazil and crude mortality is lower than 20% [12].

In terms of modality of dialysis, only 10%, of publically financed patients are currently receiving peritoneal dialysis. The fraction so treated varies tremendously across countries, with most countries having a frequency of peritoneal dialysis not too different from that of Brazil [10].

The discrete increase in the incidence of patients receiving dialysis from 2001 to 2012 was present in all regions of the country, though less so in the southeast and south, and may reflect growing access to treatment. The increase was greater in women and in the older age strata, where diabetes and hypertension present as the principal causes of ESRD. A notably larger increase in this age group was also reported in Canada a decade ago [13]. More recent series in the in the U.S. noted a larger increase in the 45 to 64 year age range [14].

The analysis of the underlying cause of ESRD in APAC is limited by the high percentage (42.3%) of diagnoses listed as indeterminate, the percentage being as high as 80% among native Brazilian patients. Among specific diagnoses listed, hypertension (20.4%) was the principal cause, followed by diabetes (12%) and then by glomerulonephritis (7.7%). These results are similar to those reported for the period of 2000-2004 by Cherchiglia *et al*. [15]. The pattern of hypertension and diabetes as predominant causes is typical of high and medium income countries, with variations in the relative positions of hypertension and diabetes. In contrast, glomerulonephritis is the more prevalent cause of ESRD in low income countries, comprising 25-35% of causes. Diabetes has been cited as the cause of ESRD in 9.1 to 29.9% of patients in different countries in the developing world, and hypertension in 13 to 21% [16]. The USRDS report shows a quite variable proportion, from 15 to 60%, of diabetes as a cause across surveyed countries [10]. Given the multicausal nature of ESRD, it is to be expected that diabetes and hypertension frequently overlap in the causal process, and this uncertainty may explain, in part, the high frequency of indeterminate cause listed in the APAC system.

Data on race/color of dialyzed patients, to our knowledge, have not been previously published in Brazil. Reported patient race/color, after redistribution of those lacking information uniformly across the race/color categories, is predominantly white (52%), followed by brown (35%), black (11%), Asian (0.9%) and native Brazilian (0.2%). These proportions are similar to the self-declared information on race/color presented in the 2010 census: white 47.7%, brown 43.1%, black 7.6%, Asian 1.0% and native Brazilian 0.4% [8].

The major strength of our study is that the database created permits analysis of a consistent series of several years of all publically-financed dialysis in Brazil, instead of relying on non-representative sampling with incomplete reporting. Aside from serving as the basis for the results here reported, this database will permit future analyses, including economic ones and those related to assessment of disease burden.

Limitations to these results merit a brief discussion. As APAC does not adequately estimate the prevalence and incidence of renal transplantation, caution is required in using these data to estimate the total number of patients receiving renal replacement therapy in Brazil, and further work is necessary to achieve estimates of ESRD incidence and prevalence. In this regard, approximately 28.4 patientspmp received a renal transplant in Brazil in 2012 [12]. As the majority of these were receiving dialysis for more than 3 months prior to receiving their transplant, underestimation of incidence of ESRD receiving renal replacement therapy is likely to be small.

Another important limitation relates to the coding of the underlying cause of renal failure, as previously noted. Similarly, though the introduction of reporting race/color of patients is an important advance, the high frequency of missing data limit precision in the reporting of this characteristic.

In summary, these analyses demonstrate the importance of the APAC system as a source for surveillance of the treatment of ESRD in Brazil. Assuming current incidence rates and lethality, the trends here presented indicate that the total number of cases needing publically financed renal dialysis will increase considerably. Given that renal replacement therapies have been reported to represent 8% of the total budget of the Ministry of Health, the projected increase will demand adequate planning of resources [17].

In terms of surveillance, continued analysis of the APAC system can provide important findings for public policies regarding the prevention, control and treatment of ESRD at local, regional and national levels. Actions and more detailed investigations should be undertaken to further qualify available data, especially with respect to the underlying causes of ESRD in Brazil. In this regard, the Brazilian Society of Nephrology has joined with the Ministry of Health in stimulating and supporting the adoption of effective measures for the surveillance, prevention, treatment and management of kidney disease in order to reduce its impact in terms of population health. The main goals within this effort are to increase awareness of risk factors for chronic kidney disease and of the importance of its complications [18].

## Conclusions

In conclusion, the epidemiologic profile for ESRD receiving dialysis in the SUS between 2000 and 2012 demonstrates a discrete yet constant increase in incidence and a larger increase in prevalence, especially in older individuals. Over this period, the number of individuals receiving renal dialysis almost doubled. These figures parallel global trends, suggesting that health care expenditures in dialysis will continue to increase in the foreseeable future, and highlight the importance of preventive measures, especially those related to the prevention and control of the main underlying causes of ESRD – hypertension and diabetes.

## Competing interests

None of the authors has any commercial association that might suggest a conflict of interest with the findings or manuscript.

## Authors’ contributions

LM, BBD and MIS participated in the conception and design of the study; LM, IVP, BBD and MIS, in data analysis; LM, BBD, FST and MIS, in data interpretation; and all authors in drafting relevant or critical revision of the intellectual content of the manuscript and final approval of the version to be published.

## Pre-publication history

The pre-publication history for this paper can be accessed here:

http://www.biomedcentral.com/1471-2369/15/111/prepub
